# Duo biologic therapy using mepolizumab and omalizumab in refractory ABPA: two cases

**DOI:** 10.1186/s13223-025-00985-0

**Published:** 2025-09-02

**Authors:** Ivan H. Huang, Kenneth N. Dang, Saarang Kashyap, Noah St. Clair, Alexander J. Sweidan

**Affiliations:** 1 Department of Physiological Science, University of California, 986 N Center Street, Orange, Los Angeles, CA 90095 USA; 2 Department of Neuroscience, University of California, Los Angeles, CA 90095 USA; 3 Department of Microbiology, Immunology & Molecular Genetics, University of California, Los Angeles, CA 90095 USA; 4 Department of Chemistry and Biochemistry, University of California, Los Angeles, CA 90095 USA; 5 Department of Pulmonary/Critical Care Medicine, University of California Irvine School of Medicine, Irvine, USA; 6 Department of Bioengineering, University of California, Los Angeles, CA 90095 USA; 7 Newport Mesa Pulmonary, Costa Mesa, USA

**Keywords:** ABPA, Biologic therapies, Mepolizumab, Omalizumab, Eosinophil, Ige, Systemic glucocorticoids, *Mycobacterium avium* complex, Asthma

## Abstract

**Background:**

Allergic bronchopulmonary aspergillosis (ABPA) presents with a wide range of symptom severity, with severe disease manifestations being harder to control through conventional inhalers. While corticosteroids remain a standard treatment option, their use is often hindered by significant adverse side effects. This case series discusses a novel treatment of duo-administration of monoclonal antibodies for two patients that reduced their exacerbations, spared the use of steroids, and improved their quality of life.

**Case presentation:**

Both patients were diagnosed with ABPA. Before the administration of treatment, they experienced almost monthly exacerbations and infections requiring constant systemic oral corticosteroids and antibiotics. After the implementation of successive concomitant monoclonal antibody treatments, absolute eosinophil levels were brought down to normal levels, and the monthly exacerbations were eliminated.

**Conclusion:**

This case series describes a novel approach for ABPA therapy that holds potential in improving patient outcomes for those with severe ABPA. Duo biologic therapy may improve disease control and reduce corticosteroid reliance in patients with refractory ABPA by targeting multiple mechanistic pathways of inflammation. Mepolizumab with Omalizumab offers a potential treatment strategy to reduce exacerbation frequency and severity and has minimal adverse effects.

## Background

Allergic Bronchopulmonary Aspergillosis (ABPA) is an immunologically mediated pulmonary disorder primarily seen in patients with asthma or cystic fibrosis, triggered by a hypersensitivity response to *Aspergillus fumigatus*. ABPA is characterized by elevated serum IgE and eosinophilia. IgE binds to high-affinity FcεRI receptors on mast cells and basophils and leads to the release of inflammatory mediators like histamine, prostaglandins, and leukotrienes as part of the IgE pathway. In parallel, interleukin-5 (IL-5) binds to its receptor on eosinophils, enhancing their activation, survival, and chemotaxis to sites of inflammation, where they release cytotoxic granules and pro-inflammatory cytokines as part of the eosinophil pathway [1]. Left untreated, it can lead to irreversible airway remodeling, causing bronchiectasis and associated infections.

The International Society for Human & Animal Mycology (ISHAM) guidelines use chronic systemic glucocorticoids to suppress chronic lung inflammation and the progression of the disease [2]. However, their use is often limited by side effects such as immunosuppression, osteopenia, hyperglycemia, steroid-induced diabetes mellitus, and muscle atrophy [3]. Antifungal agents such as itraconazole and voriconazole were previously considered first-line treatments; however, due to their significant side effects, they are now reserved for use only in patients with severe or persistent symptoms.

Biologic therapies, including anti-IgE (omalizumab) and anti–IL-5 (mepolizumab) monoclonal antibodies, have emerged as steroid-sparing options for ABPA [4]. However, the use of duo biologic therapy remains poorly documented, with only limited case reports and no consensus guidelines on its efficacy or safety. In 2017, successful dual-Omalizumab and Mepolizumab in ABPA reported dramatic reduction in IgE, eosinophils, and corticosteroid use in a patient [5].

We present two cases of refractory ABPA in which combination therapy with mepolizumab and omalizumab led to sustained clinical improvement and corticosteroid reduction.

## Case presentation

**Fig. 1:**
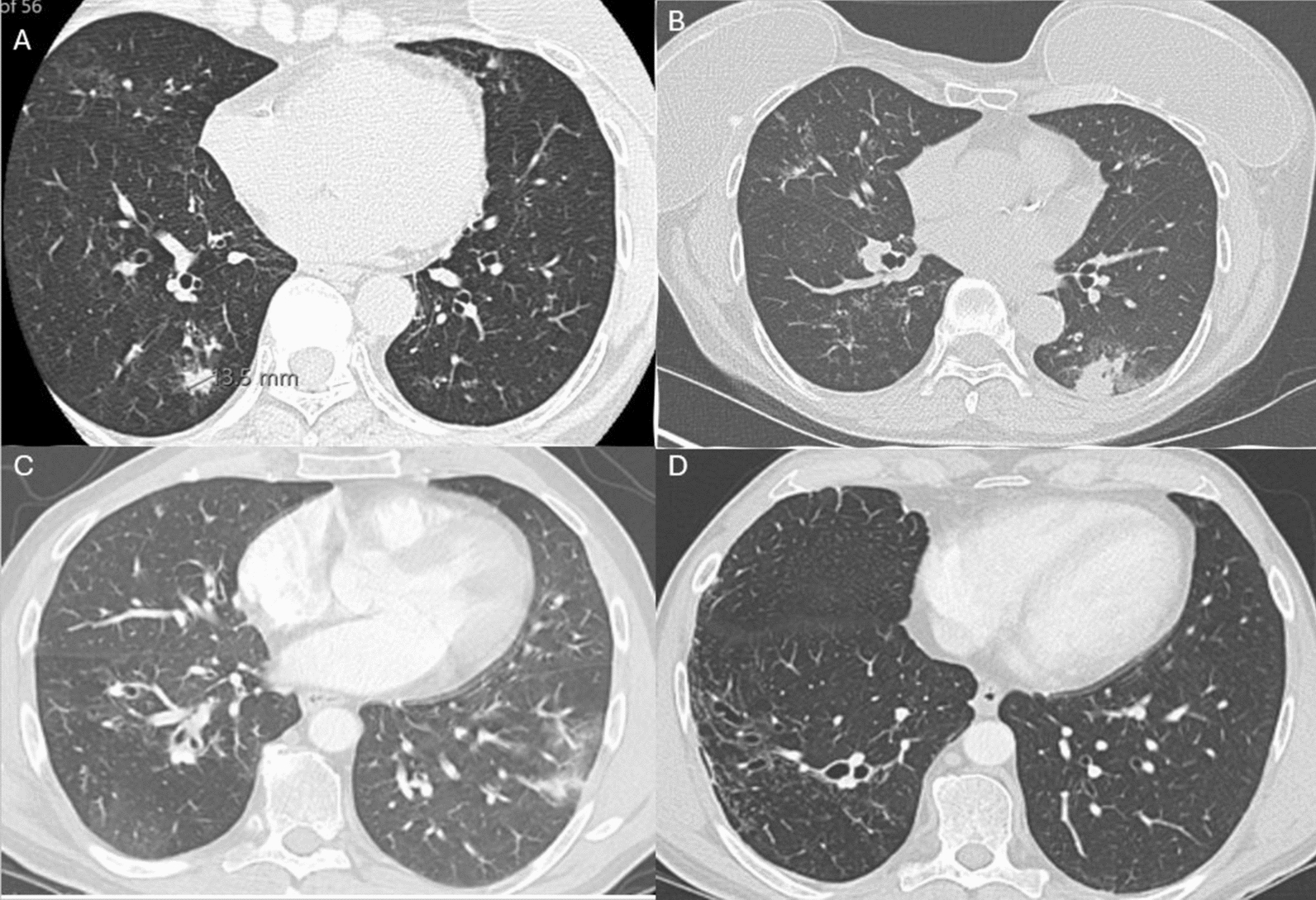
Axial chest CT images before and after dual-biologic therapy with omalizumab + mepolizumab. **A**. Patient 1 Pre-duo therapy scan reveals bilateral bronchiectasis with dense mucus impaction and patchy consolidation, most pronounced in the right lower lobe. **B**. Patient 1 follow-up scan after 6 months of duo biologic therapy shows right sided but stable mucus impaction with no progression of bronchiectasis. **C**. Patient 2 Pre-duo therapy scan shows central cylindrical bronchiectasis, mucus plugging, and mild mosaic attenuation. **D**. Patient 2 follow-up scan after 43 months of duo biologic therapy demonstrates unchanged bronchiectasis without new parenchymal injury or additional mucus burden, indicating radiographic stability despite corticosteroid withdrawal

Patient 1 is a 65-year-old female with frequent asthma exacerbations due to ABPA, elevated IgE 7699 IU/mL, and absolute eosinophils 152 cells/μL. She was initially managed with inhaled triple therapy, prednisone, and azithromycin for exacerbations, but later added mepolizumab due to corticosteroid-induced osteoporosis. On Monotherapy, she experienced two exacerbations of ABPA. IgE rose to 14,025 IU/mL while absolute eosinophil count dropped to 0 cells/μL after 13 months. Duo biologic therapy was initiated with omalizumab 375 mg and mepolizumab 100 mg every 4 weeks to control IgE-mediated inflammation. She reported significant improvement in dyspnea and chest congestion. At follow-up, IgE was 19,032 IU/mL, absolute eosinophils remaining at 0 cells/μL, FEV₁ improved from 1.36L to 1.56L. No adverse effects were reported for over 5 months. Baseline chest CT demonstrated bilateral bronchiectasis, mucus plugging, and mosaic attenuation, consistent with ABPA. Follow-up imaging after 6 months of dual biologic therapy showed no progression of bronchiectasis or parenchymal damage, indicating radiographic stability despite withdrawal of corticosteroids (Fig. 1).

Patient 2 is a 61-year-old male with hypogammaglobulinemia and asthma presenting with recurrent monthly pneumonias and empyemas, requiring chronic prednisone and VATS (video-assisted thoracoscopic surgery) decortication. He failed triple therapy, itraconazole and prednisone treatments over 3 months, and experienced steroid-induced side effects, including mania. Labs showed at IgE 2386 IU/mL and absolute eosinophils at 572 cells/μL. Mepolizumab 100 mg every 4 weeks was initiated, resulting in absolute eosinophil decreasing to 130 cells/μL, but persistently elevated IgE at 3579 IU/mL. After three pneumonia-induced asthma exacerbations, treatment with omalizumab 375 mg every 4 weeks was initiated, resulting in significant symptomatic improvement. Within 6 months of combination therapy, absolute eosinophils dropped to 19 cells/μL, and FEV₁ improved from 3.10 to 3.44 L. Even after contracting a COVID-19 infection, he reported minimal symptoms while on duo biologic (duo) therapy. He remains asymptomatic with no exacerbations and continues therapy without adverse effects. Chest CT prior to dual biologic therapy revealed central bronchiectasis, mucus plugging, and mild mosaic attenuation, consistent with ABPA-related airway remodeling. Follow-up imaging after 48 months of therapy showed reduced mucus plugging and stabilization of bronchiectasis without progression (Fig. 1).

## Discussion and conclusions

ABPA involves both eosinophilic inflammation and elevated IgE-mediated hypersensitivity, so targeting only one of these pathways may be insufficient in refractory cases, necessitating duo therapy. Mepolizumab inhibits the eosinophil pathway by binding to IL-5 to inhibit IL-5R + cell activation. This inhibits eosinophil survival, differentiation, and recruitment to inflamed tissue, preventing their activation and release of proinflammatory cytokines [6]. Omalizumab works by binding to and blocking the Cε3 locus on IgE, blocking the binding to FcεRI [7]. This prevents new sensitization events and downregulates FcεRI expression on effector cells, decreasing their reactivity over time. However, Omalizumab-bound IgE still remains in the blood with IgE levels remaining elevated but stable due to the increased half-life of Omalizumab-bound IgE. A retrospective study of 41 patients showed that while omalizumab increased total IgE levels, patients still showed significant clinical improvement [8]. In this way, Omalizumab renders free IgE biologically inactive and unable to start the IgE inflammation cascade, while Mepolizumab eliminates the eosinophil inflammation cascade.

**Fig. 2:**
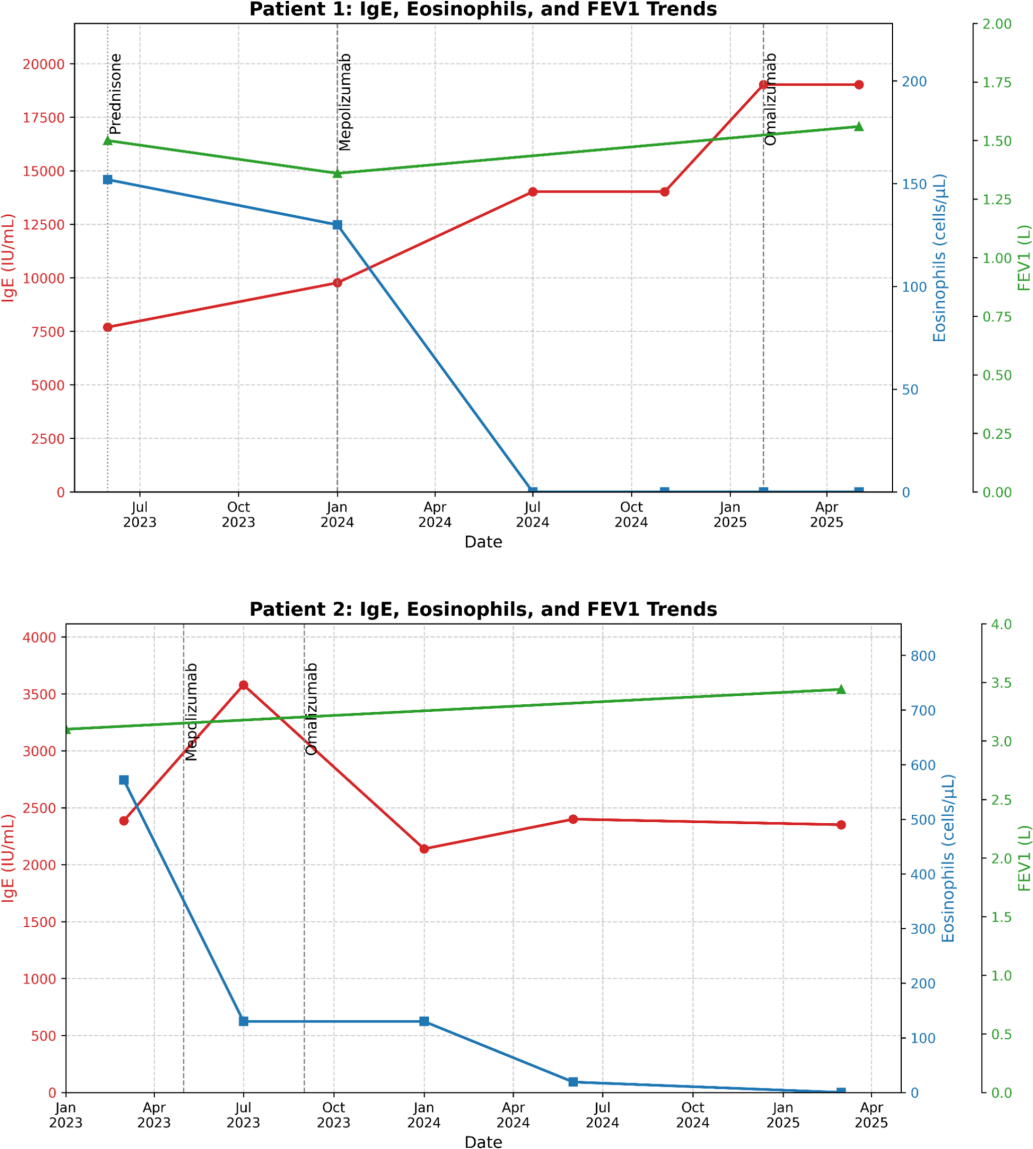
Trends in FEV1, IgE, and Absolute Eosinophils for two APBA patients following mono and duo biologic therapy

**Fig. 3:**
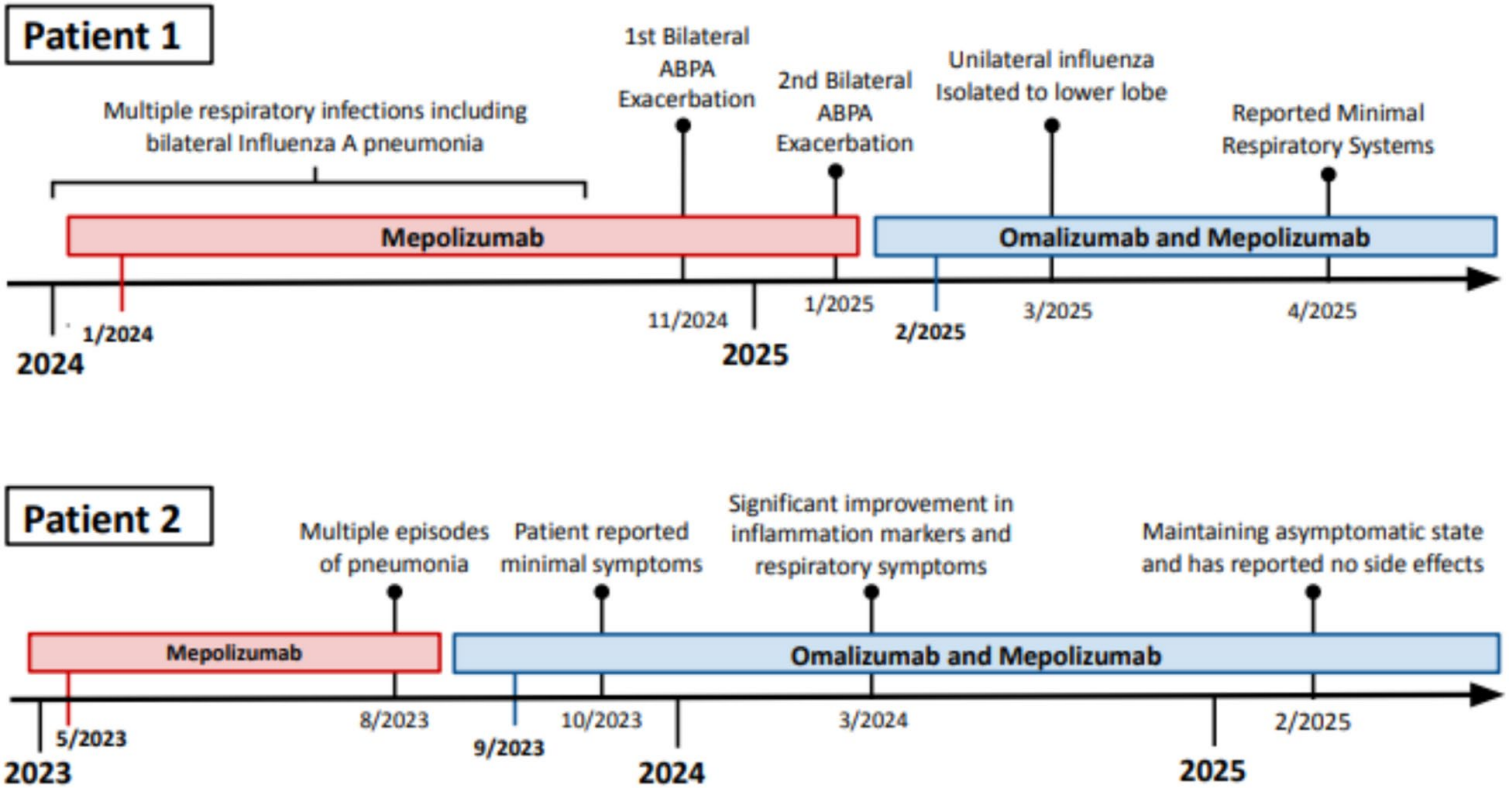
Subjective response of patient following initiation of mono and duo biologic therapy

As seen in both patients, eosinophilia was greatly reduced, and total blood IgE levels remained elevated but stable; both patients had improved symptom control and reduced infections (Table 1). Additionally, both patients showed substantially reduced exacerbations and improved respiratory function. Patient 1 and patient 2 showed a clinically meaningful FEV₁ increase of 0.200L and 0.340 L respectively, surpassing the minimal threshold of 0.158 L to see improvement (Fig. 2) [9]. Improvements in FEV₁ reflect a reduction in airway inflammation and obstruction, suggesting that treatment successfully reversed airflow limitation and improved ventilatory capacity [9]. These changes can be trended over time to track improvements and deteriorations in treatment response. In both patients, the frequency and severity of respiratory infections causing exacerbations were severely reduced after initiating duo-biologic therapy with no adverse effects reported (Fig. 3). However, the long-term safety of duo therapy, especially in immunocompromised patients or those with co-infections, warrants further study.

**Table 1:** Participant demographics and clinical characteristics at baseline, after first biologic, and after second biologic

Characteristic	Patient 1	Patient 2
Age (y)	65	61
Sex	F	M
BMI (kg/m^2^)	20.37	25.09
Atopy	Y	Y
FEV1 (L)	1.50 → 1.36 → 1.56	3.10 → 3.44
IgE (IU/mL)	7699 → 14,025 → 19,032 → 18,485	2386 → 3579 → 2351 → 2961
Eosinophils (cells/μL)	152 → 0 → 0	572 → 130 → 19 → 0
Initial Inhaler	Budesonide/Glycopyrrolate/Formoterol Fumarate	Fluticasone Furoate/Umeclidinium/Vilanterol
OCS dependence	Y	Y
First Biologic	Mepolizumab	Mepolizumab
Reason for 2nd Biologic	Persistent exacerbations	Persistent exacerbations
Second Biologic	Omalizumab	Omalizumab
Duo Biologic Doses	Omalizumab 375 mg + Mepolizumab 100 mg	Omalizumab 375 mg + Mepolizumab 100 mg
Exacerbation Frequency	Monthly Infections → Asymptomatic	Monthly Infections → Asymptomatic
Side Effects	None on duo therapy	None on duo therapy

In conclusion, combination biologic therapy using omalizumab and mepolizumab may offer significant clinical benefit in patients with severe obstructive pulmonary diseases, particularly those unresponsive to standard therapies. Duo therapy can manage severe, refractory asthma, particularly in patients exhibiting overlapping allergic and eosinophilic phenotypes, persistent symptom burden, or corticosteroid dependence despite monotherapy. Although larger studies beyond asthma and ABPA are necessary, our cases suggest duo therapy as a promising option in improving symptom control, reducing corticosteroid dependence, and minimizing exacerbation frequency and severity in high-risk patients. This approach may be particularly beneficial for complex asthma endotypes where multiple inflammatory pathways contribute to persistent disease activity and poor clinical control.

## Data Availability

No datasets were generated or analysed during the current study.

## Abbreviations

ABPA: Allergic Bronchopulmonary Aspergillosis
VATS: Video-assisted thoracoscopic surgery
